# Structure and function of the amygdaloid NPY system: NPY Y2 receptors regulate excitatory and inhibitory synaptic transmission in the centromedial amygdala

**DOI:** 10.1007/s00429-015-1107-7

**Published:** 2015-09-13

**Authors:** J. Wood, D. Verma, G. Lach, P. Bonaventure, H. Herzog, G. Sperk, R. O. Tasan

**Affiliations:** 1 Department of Pharmacology, Medical University Innsbruck, Peter-Mayr-Strasse 1a, 6020 Innsbruck, Austria; 2 Capes Foundation, Ministry of Education of Brazil, Brasília, DF 70040-020 Brazil; 3 Janssen Research & Development, LLC, San Diego, CA USA; 4 Neuroscience Division, Garvan Institute of Medical Research, Darlinghurst, Sydney, NSW 2010 Australia; 5 Institute of Physiology I (Neurophysiology), Westfälische Wilhelms-Universität, Munster, Germany

**Keywords:** Neuropeptide Y, NPY, Y2 receptor, Central amygdala, Intercalated neurons, Dopamine D1 receptor

## Abstract

The amygdala is essential for generating emotional-affective behaviors. It consists of several nuclei with highly selective, elaborate functions. In particular, the central extended amygdala, consisting of the central amygdala (CEA) and the bed nucleus of the stria terminalis (BNST) is an essential component actively controlling efferent connections to downstream effectors like hypothalamus and brain stem. Both, CEA and BNST contain high amounts of different neuropeptides that significantly contribute to synaptic transmission. Among these, neuropeptide Y (NPY) has emerged as an important anxiolytic and fear-reducing neuromodulator. Here, we characterized the expression, connectivity and electrophysiological function of NPY and Y2 receptors within the CEA. We identified several NPY-expressing neuronal populations, including somatostatin- and calretinin-expressing neurons. Furthermore, in the main intercalated nucleus, NPY is expressed primarily in dopamine D1 receptor-expressing neurons but also in interspersed somatostatin-expressing neurons. Interestingly, NPY neurons did not co-localize with the Y2 receptor. Retrograde tract tracing experiments revealed that NPY neurons reciprocally connect the CEA and BNST. Functionally, the Y2 receptor agonist PYY_3-36_, reduced both, inhibitory as well as excitatory synaptic transmission in the centromedial amygdala (CEm). However, we also provide evidence that lack of NPY or Y2 receptors results in increased GABA release specifically at inhibitory synapses in the CEm. Taken together, our findings suggest that NPY expressed by distinct populations of neurons can modulate afferent and efferent projections of the CEA via presynaptic Y2 receptors located at inhibitory and excitatory synapses.

**Electronic supplementary material:**

The online version of this article (doi:10.1007/s00429-015-1107-7) contains supplementary material, which is available to authorized users.

## Introduction

Neuropeptide Y (NPY) is a 36-amino acid peptide that is widely distributed in the central nervous system. It is particularly known for its involvement in the regulation of appetite, pain perception and maintenance of energy homeostasis, but also for its anxiolytic properties. Recently an involvement in models of conditioned fear has been also demonstrated (Broqua et al. 1995; Fendt et al. 2009; Gutman et al. 2008; Lach and de Lima 2013; Verma et al. 2012). NPY acts through at least five different G protein-coupled receptors (Y1, Y2, Y4, Y5 and y6) that in general exert a prolonged inhibitory action (Michel et al. 1998). In the CNS, postsynaptic Y1 and predominantly presynaptic Y2 receptors are the most abundant (Roder et al. 1996; Dumont et al. 1996). NPY and Y2 receptors are particularly enriched in the hippocampus and hypothalamus, but also in the central extended amygdala (Gray and Magnuson 1992; Stanic et al. 2011).

The amygdala consists of several different nuclei and controls emotional-affective behaviors such as fear and anxiety (Pape and Pare 2010; Sah et al. 2003). The central nucleus of the amygdala (CEA) is not only the major output station of the amygdala but consists itself of a highly complex micro-network capable of diverse types of plasticity (Ciocchi et al. 2010; Haubensak et al. 2010; Li et al. 2013; Wilensky et al. 2006). The CEA can be divided into at least 3 distinct subnuclei, namely the centromedial (CEm), centrolateral (CEl) and centrocapsular (CEc) nucleus. The main amygdala output originates from the CEm and consists of GABAergic neurons with efferent projections targeting different effector brain regions, including hypothalamus, brainstem and bed nucleus of the stria terminalis (BNST) (Dong et al. 2001; LeDoux et al. 1988). Interestingly, CEA neurons express high concentrations of different neuropeptides. Ample evidence suggests that these neuropeptides are significantly shaping the overall emotional response generated by the amygdala (Bowers et al. 2012; Gutman et al. 2008; Heilig et al. 1994; Heilig 2004; Tasan et al. 2010). In the BNST, Y2 receptors have been demonstrated to significantly reduce the frequency of miniature inhibitory postsynaptic currents (mIPSC), indicating their presynaptic localization on inhibitory neurons (Kash and Winder 2006).

As both, the CEA and BNST, are components of the central extended amygdala and thus exhibit considerable neuroanatomical analogies, we hypothesized that Y2 receptors in the CEA would reduce inhibitory input by a similar mechanism. To shed more light on the NPY and Y2 receptor-dependent micro-network within the CEA we utilized wildtype and various germline knock-out models, combined with slice electrophysiology, neuronal tract-tracing and immunohistochemistry. We illustrate here the exact localization as well as the afferent and efferent projections of NPY and Y2 receptor containing neurons of the CEA. Furthermore, we demonstrate that presynaptic Y2 receptors are crucially involved in the regulation of both, GABA and glutamate release onto CEm neurons.

## Experimental procedures

### Animals

All procedures involving animals and animal care were conducted in accordance with international laws and policies (Directive 2010/63/EU of the European parliament and of the council of 22 September 2010 on the protection of animals used for scientific purposes; Guide for the Care and Use of Laboratory Animals, US National Research Council, 2011) and were approved by the Austrian Ministry of Science. All efforts were taken to minimize the number of animals used and their suffering.

**Fig. 1:**
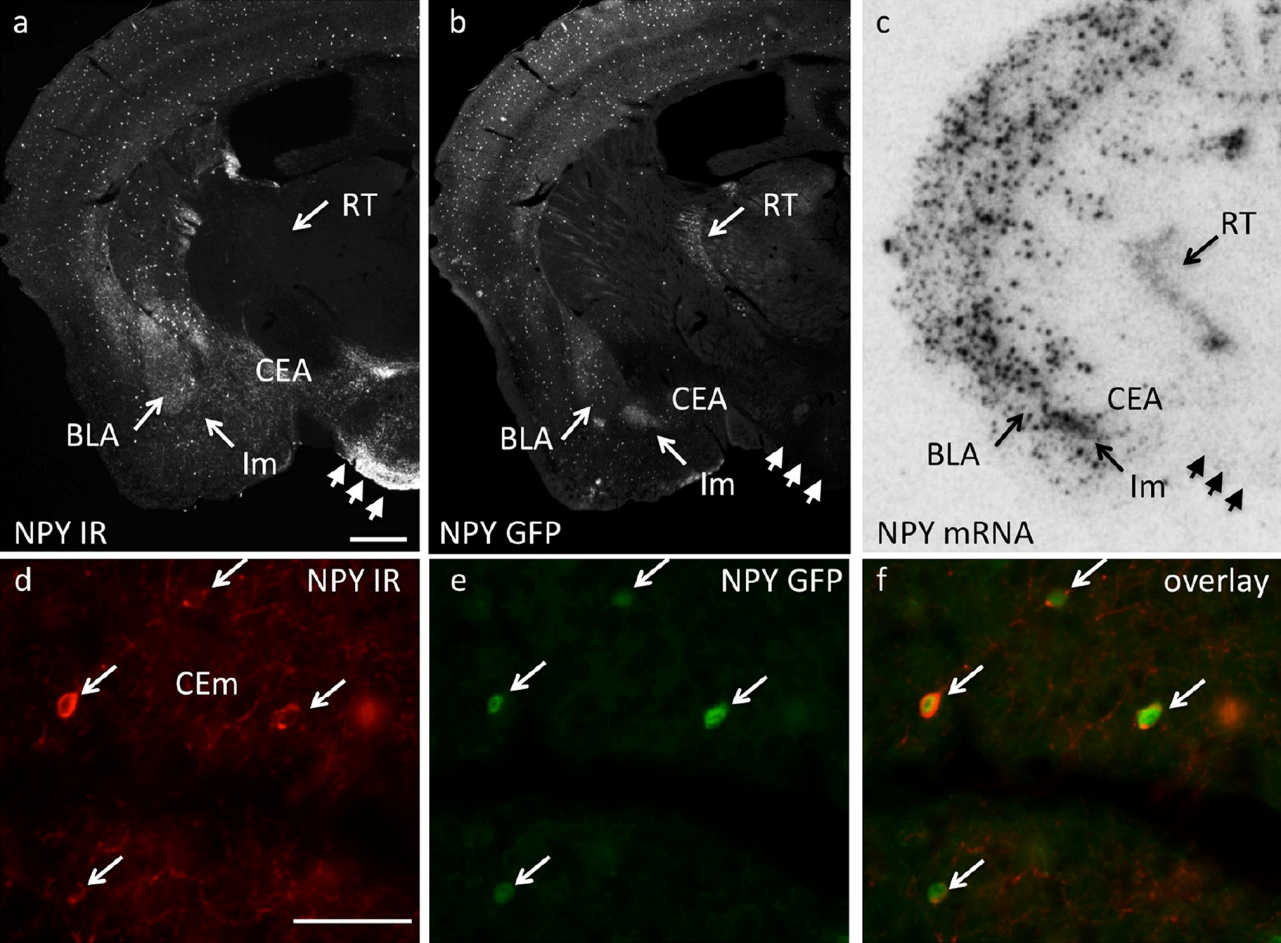
Validation of the NPY-GFP mouse. **a** Photomicrograph of NPY immunoreactivity and **b** NPY-GFP immunoreactivtiy in coronal brain sections of an NPY-GFP mouse compared to, **c** autoradiograph of an in situ hybridization for NPY mRNA, demonstrating an overlapping distribution pattern of NPY mRNA and NPY-GFP. Dual immunohistochemistry exemplified in the CEm for **d** NPY, **e** NPY-GFP and **f** overlay demonstrates extensive co-localization (note the *arrows* in **a**–**c** displaying similar distribution of cell bodies in the basolateral amygdala (*BLA*), main intercalated nucleus (*Im*) and reticular thalamic nucleus (*RT*) of NPY-GFP and NPY mRNA labeling compared to NPY-IR that is frequently confined to axons and axon terminals—*arrowheads*). *Scale bars* **a**–**c** 1 mm, **d**–**f** 100 µm

All experiments were performed in adult male mice (10–16 weeks old, weighing 25–30 g) maintained on a pure C57BL/6N background (Charles River, Sulzfeld, Germany). Germline knock-out mice for the Y2 receptor (Y2KO) as well as NPYKO mice were backcrossed to a C57BL/6N background for at least 10 generations. Mice were housed in groups of 3–5 animals under standard laboratory conditions (12 h/12 h light/dark cycle, lights being on at 07:00, food and water ad libitum). Generation of NPYKO mice and Y2KO mice has been described in detail previously (Sainsbury et al. 2002; Verma et al. 2012). For immuolabeling procedures and electrophysiological recordings specifically from NPY neurons, we used an NPY-GFP mouse line that expresses GFP from the NPY promoter (B6.FVB-Tg(Npy-hrGFP)1Lowl/J). This transgenic mouse line was characterized previously (van den Pol et al. 2009) and we confirmed the identity of GFP neurons by dual immunofluorescence of hrGFP and endogenous NPY (Fig. 1).

### Electrophysiology

#### Acute brain slice preparation

Adult male mice were anesthetized with isoflurane (Baxter, Austria), decapitated and the brain was rapidly removed, hemisected and placed in ice-cold oxygenated (95 % O_2_/5 % CO_2_) artificial cerebrospinal fluid (aCSF) containing (in mM) 126 NaCl, 2.5 KCl, 1.25 NaH_2_PO_4_, 1 MgCl_2_, 26 NaHCO_3_, 2 CaCl_2_ and 10 glucose. Coronal brain slices (300 μm thick) containing the CEm (3–4 sections per mouse) and Im (1–2 sections per mouse) were cut using a vibratome (VT1200, Leica Microsystems, Germany). Slices were allowed to recover for at least 1 h in aCSF before being transferred to a recording chamber constantly perfused with aCSF and gradually warmed to the recording temperature of 32–34 °C.

#### Electrophysiological recordings

For whole-cell voltage-clamp recordings (holding potential = −70 mV) of inhibitory and excitatory postsynaptic currents (IPSCs and EPSCs, respectively), recording pipettes with a final tip resistance of 2–5 MΩ were pulled using a micropipette puller (P-1000, Sutter Instrument, USA) and filled with solution containing (in mM) 135.0 CsCl, 10.0 CsOH-HEPES, 0.2 CsOH-EGTA, 2.0 Mg-ATP, 0.3 Na_3_-GTP, 8.0 NaCl and 5.0 lidocaine N-ethyl bromide (QX-314) for sIPSCs; or 117.5 Cs-gluconate, 17.5 CsCl, 8.0 NaCl, 10.0 CsOH-HEPES, 0.2 CsOH-EGTA, 2.0 MgATP, 0.3 Na_3_-GFP and 5.0 QX-314 for sEPSCs. For whole-cell current-clamp recordings (holding current = 0 pA), the pipette solution contained (in mM) 122.5 K-gluconate, 12.5 KCl, 10.0 KOH-HEPES, 8.0 NaCl, 0.2 KOH-EGTA, 2.0 MgATP and 0.3 Na_3_GTP. Im neurons were identified using NPY-GFP mice (see Fig. 3). Evoked inhibitory postsynaptic currents were measured in voltage-clamp configuration in response to electrical stimulation (20–150 μA: set to the minimum current required to consistently evoke responses, i.e., >90 % success rate) of medial inputs to the CEm. Electrical stimulations were delivered using a concentric, bipolar platinum/iridium electrode with a 2–3 μm tip diameter (MicroProbes, USA) connected to a constant current stimulator (Digitimer, UK). Neurons of the CEm were visually identified based on their anatomical location using an upright microscope (BX51, Olympus, Japan) equipped with a 40× water immersion objective, infrared light with differential interference contrast and a digital camera. For recordings of IPSCs AMPA and NMDA glutamate receptors were blocked using 10 μM 6,7-dinitroquinoxaline-2,3-dione (DNQX) and 100 μM DL-2-amino-5-phosphonopentanoic acid (DL-AP5; Abcam, UK), respectively. GABA_A_ receptors were blocked with 100 μM picrotoxin (Tocris Bioscience, UK). PYY_3-36_ (50–100 nM, Polypeptide, Strasbourg, France) and JNJ-31020028 (1 μM, Janssen Research & Development, LLC, San Diego, USA) were dissolved in aCSF containing DL-AP5 and DNQX or picrotoxin and administered by bath application. Compared to NPY_3-36_, PYY_3-36_ shows similar affinity and specificity for Y2 receptors, it is, however, less prone to adsorption to the tubing and thus better suited for slice electrophysiology experiments. Nonetheless, only 1 cell was recorded per slice in case complete washout could not be achieved. The D1 receptor agonist (A68930, 500 nM, Tocris, UK) was dissolved in aCSF containing DL-AP5, DNQX and picrotoxin. Cells exhibiting >20 % changes in access resistance or holding current were excluded from analysis. Data were filtered at 2.9 kHz and sampled at 10 kHz with an EPC10 patch-clamp amplifier and analyzed using PatchMaster and FitMaster software (HEKA Electronic, Germany) and Minianalysis (Synaptosoft, USA). For analysis of sIPSCs and sEPSCs, baseline events were recorded for 2 min prior to PYY_3-36_ and JNJ-31020028 application and compared with the final 2 min of drug application (4–6 min after wash-in). All events from the respective time periods were analyzed and averaged for each cell.

### Neuronal tract tracing

To identify NPY containing neurons that reciprocally connect CEm and BNST, the retrograde neuronal tracer hydroxystilbamidine (1 % Fluorogold, FG, Fluorochrome LLC, USA) was injected unilaterally into the CEm or BNST of deeply anesthetized, male, 8–12 weeks old NPY-GFP mice (3 NPY-GFP mice for CEA injections and 3 NPY-GFP mice for BNST injections). Neuronal tracers (0.2 µl) were pressure injected by glass cannulas connected to a pneumatic pressure-injector (TooheySpritzer, Science products, Germany) using the following coordinates for the CEA and BNST (in mm, from bregma): CEA: A, −1.0; L, ±2.8; V, −4.9; BNST: A, 0.0; L, ± 1.0; V, −4.6. One week after injections, mice were deeply anesthetized by a lethal dose of thiopental (Thiopental, Sandoz, Austria) and transcardially perfused with ice-cold 4 % PFA (10 min, 9 ml/min). Brains were postfixed for 90 min in the same solution, cryoprotected by immersion into 20 % sucrose (24 h) and snap-frozen in isopentane (−70 °C, 3 min).

### Histochemistry

For in situ hybridization and receptor autoradiography 20 µm coronal sections from snap-frozen mouse brains were used.

#### In situ hybridization

Oligonucleotides (2.5 pmol) were 3′ end-labeled using [^35^S]α-dATP (50 µCi; 1300 Ci/mmol, Hartmann Analytic GmbH, Braunschweig, Germany) with terminal transferase (Roche Diagnostics, Basel, Switzerland) and incubated on 20 µm coronal brain sections, as described previously in detail (Tasan et al. 2010, 2011).

#### Y2 receptor autoradiography

C-terminally truncated human peptide YY (hPYY_3-36_) was radiolabeled with Na^125^I (2200 Ci/mmol; PerkinElmer, Boston, USA), and brain sections were incubated with the radiolabeled peptide as described in detail previously (Tasan et al. 2009).

#### Tissue preparation

For immunohistochemical co-labeling in total 8 NPY-GFP mice were used. Mice were killed by injecting a lethal dose of thiopental (Thiopental, Sandoz, Austria) and brains were perfused with 4 % paraformaldehyde (PFA) for immunohistochemistry (Tasan et al. 2010).

#### Immunohistochemistry

Antibodies for NPY and somatostatin were produced in-house and have been validated previously (Bellmann et al. 1991; Sperk and Widmann 1985). Furthermore, a detailed characterization for the NPY-GFP mouse and the respective hrGFP antibody (Fig. 1) as well as antibodies for the Y2 receptor (Fig. 4) and GABA (Supplementary Fig. 1) is included in this study. Immunohistochemical analyses were performed on free-floating, PFA-fixed, 40-µm-thick coronal sections using indirect peroxidase labeling, as described previously (Tasan et al. 2011). In brief, coronal sections were incubated free floating in 10 % normal horse or goat serum (Biomedica, Vienna, Austria) in Tris–HCl buffered saline (TBS; 50 mM, pH 7.2) for 90 min, followed by incubation with primary antiserum (Table 1). The resulting complex was visualized by incubation with horseradish peroxidase (HRP)-coupled secondary antibody (1:250 P0448; Dako, Vienna, Austria) at room temperature for 150 min. For immunofluorescence sections were incubated in a tyramide signal amplification solution (1:100, TSA fluorescein, in-house) for 3–8 min. After staining, sections were exposed to 0.01 M HCl for 20 min at room temperature to denature HRP and antibodies or incubated with 3 % H_2_O_2_ to denature HRP peroxidase followed by incubation with a second antibody as described before except that staining was performed with TSA AMCA (1:100, in-house). NPY-GFP was visualized by endogenous fluorescence or by a secondary antibody conjugated to an Alexa-Fluor 488 dye (Molecular Probes, A21206, 1:1000). Sections were mounted on slides and covered using Vectashield mounting medium (Vector laboratories, Inc., Burlingame, USA).

**Table 1:** List of primary antibodies

Primary antibody	Species	Code	Source	Characterization	Dilution
Calretinin	Goat	AB1550	Chemicon	WB, IHC	1:500
Calretinin	Rabbit	7697	Swant	WB, IHC, no labeling in knock-out mice	1:1000
Dopamine 1 receptor	Goat	Af1000	Frontier Institute, Japan	WB, IHC, Narushima et al. (2006)	1:1000
Fluorogold	Rabbit	AB153	Millipore	IHC, no labeling in non-injected mice (this study)	1:2000
GABA	Rabbit	A2052	Sigma-Aldrich	IHC, dual labeling with GAD67, Comparison to in situ hybridization for GAD67 (this study)	1:1000
Glutamate decarboxylase GAD67	Mouse	MAB5406	Chemicon	WB, IHC, comparison to in situ hybridization (this study)	1:5000
Humanized *Renilla reniformis* GFP	Rabbit	240141	Agilent	IHC, dual IHC with NPY in NPY-GFP mice, no labeling in wild-type mice (this study)	1:200
Neuropeptide Y	Rabbit	(1-5)	Bellmann et al. (1991)	HPLC, RIA, IHC, no labeling in knock-out mice	1:2000
Somatostatin	Rabbit	14 (2-5)	Sperk et al. (1985)	HPLC, RIA, IHC	1:2000
Y2 receptor	Rabbit	RA14112	Neuromics	IHC, comparison to in situ hybridization and receptor binding, no labeling in knock-out mice (this study)	1:2000

*WB* Western blot, *IHC* immunohistochemistry, *HPLC* high-performance liquid chromatography, *RIA* radio-immuno assay

### Quantification of immunohistochemical labeling

Analysis of dual labeling immunofluorescence was done as described elsewhere (McDonald and Mascagni 2010; Tasan et al. 2011). In brief, photomicrographs were taken on a fluorescent microscope (Zeiss Axio Imager M1) equipped with a halogen light source, respective filter sets and a Hamamatsu monochrome camera (Hamamatsu ORCA ER C4742-80-12AG). The numbers of NPY-GFP, SST, CR and FG labeled cells were obtained bilaterally from 3–4 sections per animal depicting the central amygdala or BNST at a magnification of 400 times in multiple separate fields. Results are presented as total numbers and percentages of NPY-GFP-positive, single- and dual-labeled cells.

### Statistical analysis

Data are presented as mean ± SEM. They were analyzed for normal distribution and equal variances using GraphPad Prism software (Prism 5 for Macintosh, GraphPad Software Inc., San Diego, CA). Electrophysiological data were analyzed using the paired *t* test, one-way or two-way ANOVA followed by Bonferroni post hoc test.

## Results

### Distribution of NPY and Y2 receptors in the central extended amygdala and intercalated neurons

**Fig. 2:**
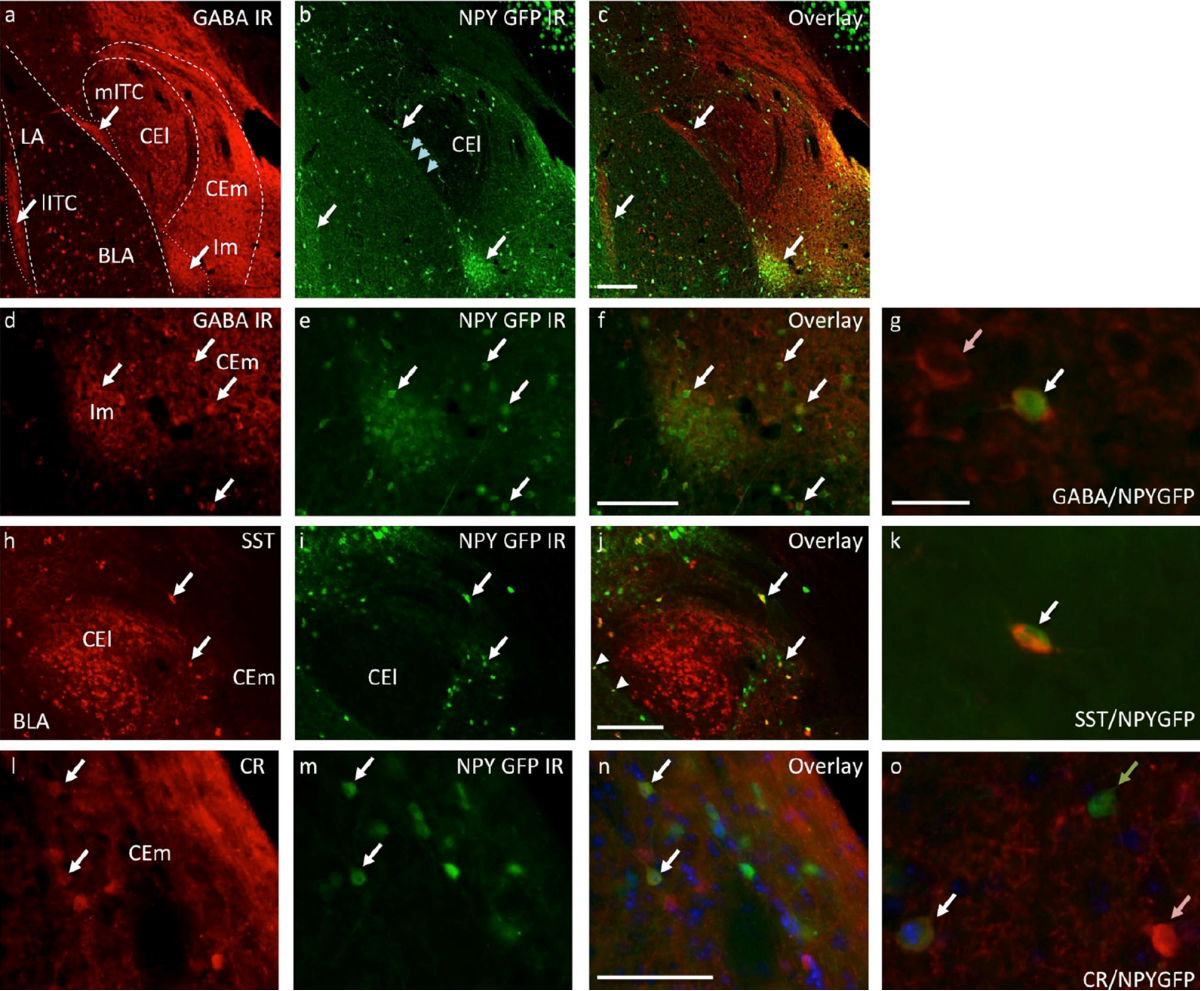
Expression of NPY in different neurons of the CEA. **a**–**c** NPY-GFP expression is evident in GABAergic neurons of the basolateral (*BLA*) and centromedial amygdala (*CEm*) and also in the lateral (*lITC*) and medial intercalated cells (*mITC*) and particularly in the main intercalated nucleus (*Im*). Note the NPY-GFP-positive fibers connecting the mITC and Im (*blue* *arrowheads*). **d**–**f** Higher magnification of NPY-GFP neurons in the Im and CEm co-localized with GABA and **g** higher magnification of a dual-labeled GABA/NPY-GFP neuron (*white* *arrow*) in the CEm (a single-labeled GABA neuron is marked with a *red* *arrow*). Note the dense, multi-layered accumulation of NPY-GFP neurons in the Im. **h**–**j** The majority of NPY-GFP neurons in the CEm co-express somatostatin (*SST*). Note the relative absence of NPY-GFP in the centrolateral amygdala, CEl and **k** higher magnification of a dual-labeled SST/NPY-GFP neuron in the CEm (*white* *arrow*). **l**–**n** An additional group of NPY neurons (*green*) in the CEm co-expresses the calcium-binding protein, calretinin (*red*) (nuclear staining in blue (Hoechst)) and **o** higher magnification of a dual-labeled CR/NPY-GFP neuron (*white* *arrow*) and NPY-GFP (*green* *arrow*) and CR (*red* *arrow*) single-labeled neurons. *White* *arrows* indicate examples of dual-labeled neurons. *Scale* *bars* **a**–**c**, **d**–**f**, **h**–**j**, **l**–**m** 200 µm and in **g**, **k**, **o** 20 µm

Since NPY is predominantly expressed in axons and axon terminals, we used a transgenic mouse line [B6.FVB-Tg(Npy-hrGFP)1Lowl/J] that expresses GFP in somata of NPY-expressing neurons. This mouse line was characterized previously (van den Pol et al. 2009) and we confirmed the validity for this study by dual immunofluorescence of hrGFP and endogenous NPY. As shown in Fig. 1, the overall distribution of NPY-IR and NPY-GFP was highly similar (Fig. 1a, b) exhibiting extensive co-localization, here exemplified in a higher magnification photomicrograph for the CEA (Fig. 1d–f). Importantly, labeling of NPY-GFP cell bodies corresponded better to the in situ hybridization for NPY mRNA than immunoreactivity with an NPY antibody (Fig. 1a–c), consistent with the expression of the NPY peptide in axons and axon terminals compared to NPY-GFP expression in cell bodies (note the highly similar expression of NPY mRNA and NPY-GFP, but only weak labeling of NPY-IR in the reticular thalamic nucleus (RT) and in the main intercalated nucleus of the amygdala (Im) as well as a higher number of NPY-GFP cell bodies in the basolateral amygdala (BLA), arrows in Fig. 1a–c). Virtually all NPY-GFP-positive neurons in the BLA, CEA and adjacent intercalated neurons were co-localized with the inhibitory neurotransmitter GABA (Fig. 2a–g), confirming that NPY in the amygdala is expressed predominantly by GABAergic neurons. The specificity of the GABA antibody has been demonstrated previously by us and others (Busti et al. 2011; Menegola et al. 2008; Tasan et al. 2011) and the immunohistochemical protocol for the present study was validated by comparing GABA immuno-labeling to an in situ hybridization for the GABA synthesizing enzyme GAD67 (Supplementary Fig. 1a, b) as well as by dual immunohistochemistry for GAD67 and GABA (Supplementary Fig. 1c–e).

### NPY in the CEA

As shown in Fig. 2, NPY-GFP-positive neurons and dense fiber staining were observed predominantly in the centromedial amygdala (CEm), with only few neurons and fibers in the centrolateral part (CEl, Fig. 2b, e, i). A considerable portion of NPY-GFP-positive perikarya in the CEA was co-localized with somatostatin (SST) (Fig. 2h–k). As shown in Table 2, 41.5 % (139/335), 35.4 % (35/99), and 49.0 % (102/208) of NPY-GFP neurons were also immuno-positive for SST in the CEm, CEc and CEl, respectively.

**Table 2:** Co-localization of NPY-GFP and SST in subdivisions of the CEA

Nucleus	Single-labeled NPY-GFP+ neurons	Single-labeled SST+ neurons	Double-labeled neurons	Percent of double-labeled NPY-GFP+ neurons	Percent of double-labeled SST+ neurons
Centrolateral	106	356	102	49.0 % (102/208)	22.3 % (102/458)
Centromedial	196	628	139	41.5 % (139/335)	18.1 % (139/767)
Centrocapsular	64	255	35	35.4 % (35/99)	12.1 % (35/290)

However, some NPY-expressing neurons in the CEm co-localized with the calcium-binding protein, calretinin (CR) (Fig. 2l-o). As summarized in Table 3, 3.4 % of the NPY-GFP neurons (16/476) in the CEm co-labeled for CR. The number of CR neurons in the CEc and CEl was not determined because dense fiber labeling made a discrimination of CR-positive cell bodies impossible. Interestingly, CR and NPY, but not SST-positive fibers were also observed in the stria terminalis, the main output fiber tract originating from the CEm, suggesting the expression of NPY on projection neurons and local neurons.

**Table 3:** Co-localization of NPY-GFP and CR in the subdivisions of the CEA

Nucleus	Single-labeled NPY-GFP+ neurons	Single-labeled CR+ neurons	Double-labeled neurons	Percent of double-labeled NPY-GFP+ neurons	Percent of double-labeled CR+ neurons
Centrolateral	n.d.	n.d.	n.d.	n.d.	n.d.
Centromedial	460	382	16	3.4 % (16/476)	4.0 % (16/398)
Centrocapsular	n.d.	n.d.	n.d.	n.d.	n.d.

*n.d*. not done, because the calretinin antibodies labeled there only fibers but no cell bodies

### NPY in the intercalated nuclei

**Fig. 3:**
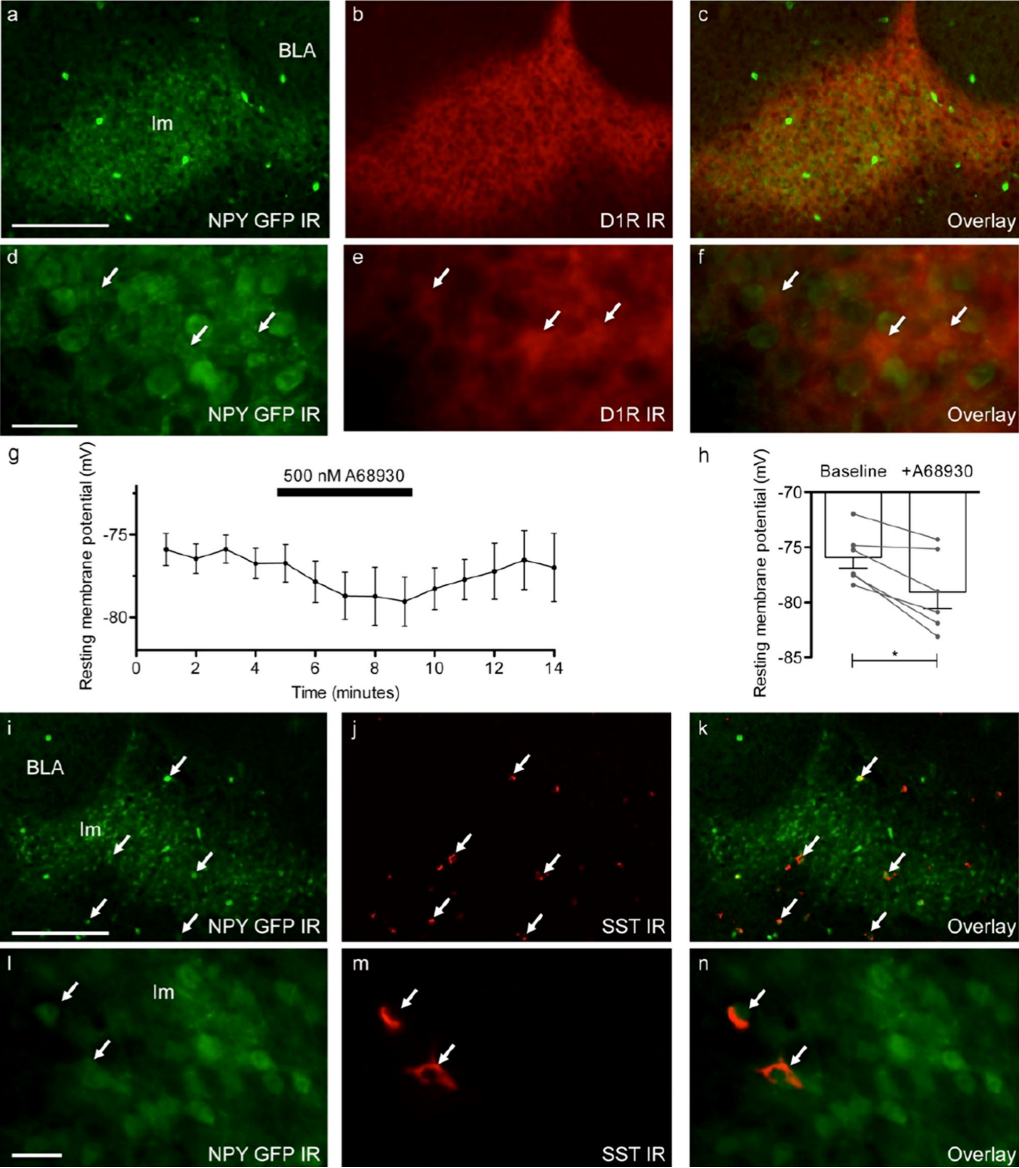
Expression of NPY in the main intercalated nucleus. **a**–**c** NPY-GFP immunoreactivity indicates at least two types of NPY neurons within the, *Im*: the majority of neurons exhibit relatively low levels of NPY expression while a second group consists of predominantly bipolar neurons with higher NPY expression. **a**–**f** The majority of NPY-expressing neurons in the Im seem to express also dopamine D1 receptor (D1R) (note the dense packing of NPY-GFP neurons in the Im that makes identification of individual neurons difficult). **d**–**f** Higher magnification image depicting NPY-GFP expression in the soma and dendrites and D1R expression frequently co-localizing with NPY-GFP-positive somata and dendritic fibers, examples are indicated by *arrows*. **g**–**h** whole-cell current-clamp recordings obtained in the presence of glutamate and GABA receptor antagonists (DL-AP5, DNQX and picrotoxin, to block synaptic transmission) demonstrated that application of the D1R agonist, A68930, reversibly hyperpolarizes Im neurons (paired *t* test: *t*_(5)_ = 4.16, *p* < 0.01; *n* = 6 cells from 6 slices from 5 mice). **i–k** Dual immunohistochemistry for NPY-GFP and SST illustrating a second group of NPY-expressing neurons in the Im. **l**–**n** Higher magnification of dual-labeled SST/NPY-GFP neurons that are located within and around the NPY-GFP cell cluster of the Im. *Scale* *bars* **a**–**c**, **j**–**k** 200 µm and **d**–**f**, **l**–**n** 20 µm

Intercalated neurons are a group of GABAergic neurons surrounding the BLA and CEA (Busti et al. 2011). These cell clusters send considerable projections to different CEA subdivisions and are considered to have a crucial role in fear and extinction learning (Amano et al. 2010; Verma et al. 2015). Thus, we also analyzed the expression of NPY and Y2 receptors in intercalated nuclei that may constitute a potential source of NPY-ergic afferences to the CEA. Interestingly, a distinct distribution was observed in the intercalated nuclei. The main intercalated nucleus (Im) was heavily populated by NPY-GFP-positive neurons, and considering the different expression levels of NPY-GFP, consisted of at least two populations of NPY-expressing neurons (Fig. 3a). The vast majority of NPY-expressing neurons in the Im were also positive for the dopamine D1 receptor (D1R) (Fig. 3a–f), while only a few, interspersed NPY-expressing neurons contained SST (i–n). Interestingly, compared to SST neurons, D1R-expressing neurons of the Im exhibited lower expression of NPY-GFP (Fig. 3a, i). Furthermore, NPY-GFP cells in the Im were densely packed and in our 40-µm sections organized in multiple overlapping layers making reliable quantification impossible. Thus, to corroborate our finding that the D1R is expressed on NPY-expressing neurons of the Im, we obtained electrophysiological recordings from GFP-expressing Im neurons in brain slices prepared from NPY-GFP mice. Whole-cell current-clamp recordings targeting GFP-expressing Im neurons demonstrated that bath application of the selective D1R agonist, A68930, resulted in a marked and reversible hyperpolarization of the resting membrane potential from −75.90 ± 1.0 mV to −79.05 ± 1.5 mV (Fig. 3g–h), indicating that the D1R is expressed postsynaptically by neurons of the Im and consistent with a previous study showing that dopamine hyperpolarizes medial and lateral intercalated cells (Marowsky et al. 2005). On the other hand, in the medial (mITC) and lateral (lITC) clusters only a few NPY-expressing neurons were discovered (Fig. 2a–c).

### Y2 receptors in the CEA

**Fig. 4:**
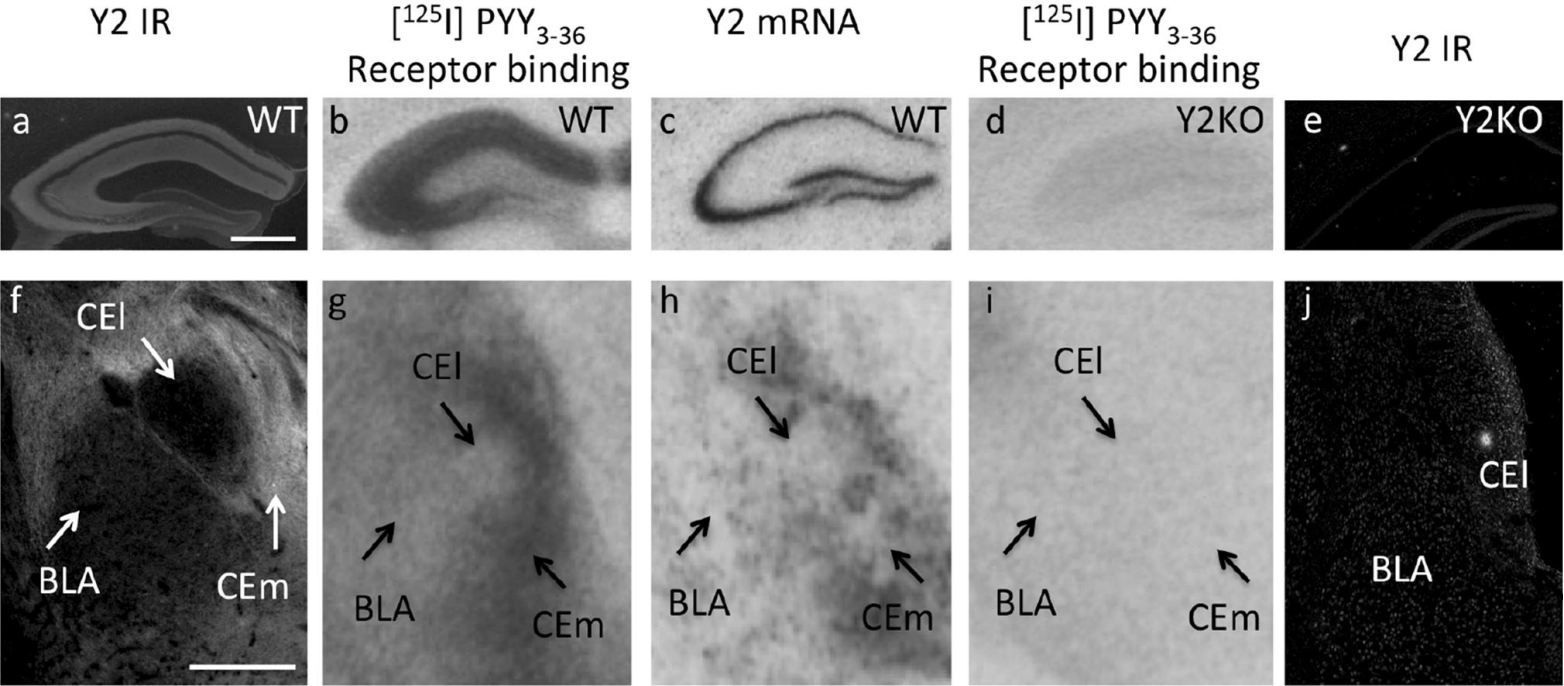
Validation of Y2 receptor antibody and immunohistochemistry procedure. **a**, **e** Photomicrograph of a Y2 receptor immunohistochemistry on a coronal section of a mouse brain depicting the dorsal hippocampus and the amygdala displays similar distribution as **b**, **f** autoradiograph of a receptor binding with the Y2 preferring agonist [^125^I]PYY_3-36_. **c**, **g** Corresponding in situ hybridization for Y2 receptor mRNA demonstrating compatible distribution with Y2 receptor immunohistochemistry and supporting the presynaptic expression of Y2 receptors. **d**, **h** However, absence of Y2 receptor binding and immunohistochemical labeling for Y2 receptors in a Y2KO mouse. (Note the unspecific staining of nuclei and somata in the Y2KO mouse frequently misinterpreted as Y2 receptor labeling). *Scale* *bars* 500 µm

**Fig. 5:**
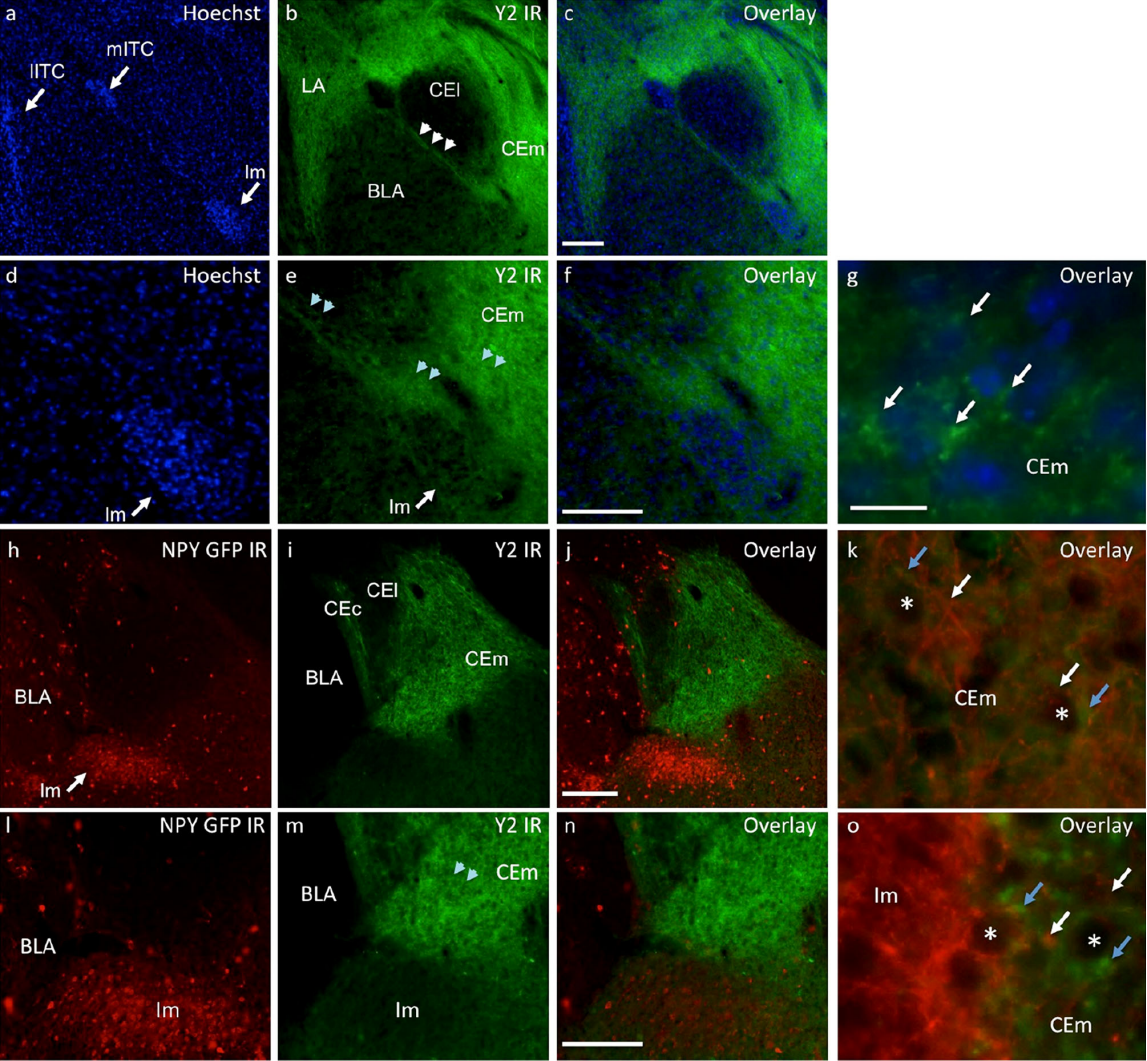
Expression of Y2 receptors (*green*) and NPY (*red*) in the central (CEA) and basolateral amygdala (BLA). **a**–**c** Dual labeling of Y2 receptor and nuclear staining (*blue*, Hoechst 33342) in the CEA and BLA. Y2 receptors are expressed predominantly in the centromedial (*CEm*), but not in the centrolateral amygdala (*CEl*). Note the relative absence of Y2 receptor expression in the lateral (*lITC*) and medial (*mITC*) intercalated cells compared to the high expression in the main intercalated nucleus (Im). Similar to NPY-GFP expression, Y2 receptor expressing fibers connect the mITC and Im (*arrowheads*). **d**–**f** Y2 receptor expression is shown in afferent and efferent connections of the Im at higher magnification, nuclear staining in blue (Hoechst 33342) and **g** higher magnification image demonstrating that Y2 receptor immuno-positive puncta are surrounding the *blue* cell nuclei in the CEm. **h**–**j** Dual labeling of NPY-GFP and Y2 receptor immunoreactivity in the anterior part of the CEA, note the high expression of NPY-GFP in the Im and the absence of co-labeling of Y2 receptor and NPY-GFP. **k** High magnification photomicrograph demonstrating that Y2 receptors (*blue* *arrows*) and NPY-GFP (*white* *arrows*) do not co-localize in the same fibers but rather Y2 receptors are bordering cell somata (*asterisks*) and are surrounded by NPY-GFP fibers. **l**–**n** Higher magnification depicting non-overlapping expression of NPY-GFP and the Y2 receptor in the Im (Abbreviation: CEc, capsular nucleus of the central amygdala) and **o** higher magnification image of the border between Im and CEm with high expression of NPY-GFP and Y2 receptors in the Im and CEm, respectively. Note that immuno-labeling for Y2 receptors (*blue* *arrows*) and NPY-GFP (*white* *arrows*) are surrounding cell bodies (*asterisks*), but do not co-localize. *Scale bars* **a**–**c**, **d**–**f**, **h**–**j**, **l**–**m** 100 µm and in **g** for **g**, **k**, **o** 20 µm

As shown in Fig. 4, the Y2 receptor antibody exhibited a similar pattern of distribution as radioactive receptor binding using the Y2 receptor preferring ligand [^125^I]PYY_3-36_ and was comparable to in situ hybridization for Y2 mRNA, emphasizing the presynaptic expression of Y2 receptors. Importantly, in Y2KO mice receptor binding with [^125^I]PYY_3-36_ as well as Y2 receptor immunoreactivity was absent (Fig. 4d, i and e,j), confirming the specificity of the Y2 receptor immunohistochemistry protocol employed in our experiments (note the residual labeling of cell nuclei and somata in Y2KO mice frequently misinterpreted as Y2 receptor immunolabeling, in Fig. 4e, j). Similar to NPY, Y2 receptor immunoreactivity (IR) was dense in the CEm, but virtually absent from the CEl (Fig. 5a–f). Interestingly, cell nuclei stained with Hoechst were surrounded by Y2R-IR (Fig. 5g), suggesting Y2 receptor containing terminals contact CEm somata. No co-localization with NPY-GFP was observed (Fig. 5h–o). At higher magnification, a clear distinction between NPY-GFP and Y2R containing fibers was observed; however, both were allocated around cell bodies in the CEm (Fig. 5k, o). Moreover, intense Y2 receptor positive fiber staining was contrasting with relatively few NPY-GFP positive fibers in the CEm (Fig. 5h–j), further suggesting that Y2 receptors are expressed by a higher number of neurons and fibers in the CEm than its corresponding ligand NPY.

### Y2 receptors in the intercalated nuclei

Consistent with a presynaptic localization, Y2 receptor IR was absent from the mITC and lITC (Fig. 5a–c) but weakly expressed in the Im and strongly expressed in the adjacent area of the CEm, the main projection target of the Im (Fig. 5i, m). Interestingly, Y2 receptor positive fibers were visible at the border between the BLA and CEA probably connecting there the mITC with the Im (arrowheads in Fig. 5b).

**Fig. 6:**
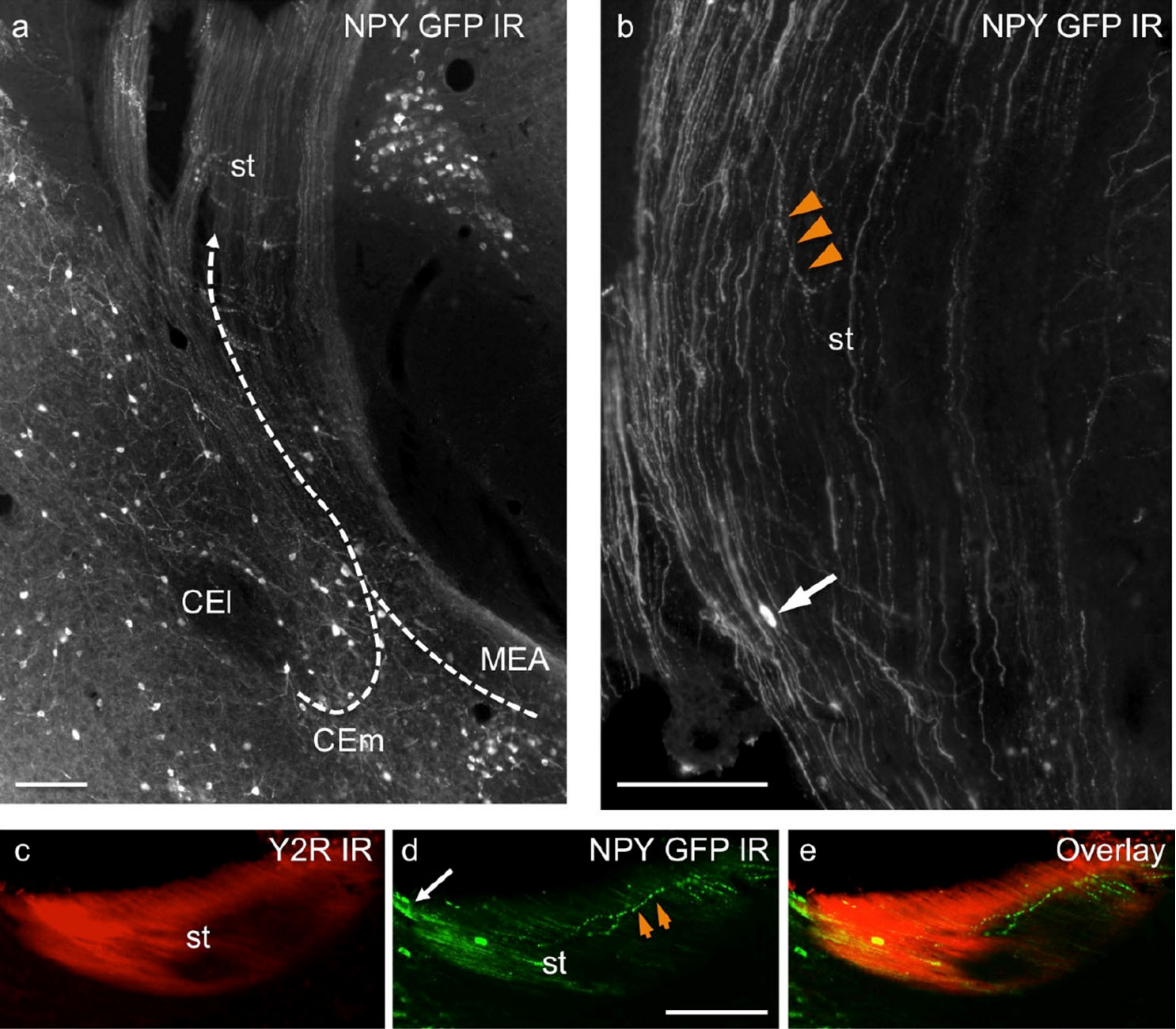
Expression of NPY in the stria terminalis. **a** NPY-expressing neurons are present in the CEm/BNST, note the high density of NPY-positive fibers in the stria terminalis. **b** Two different types of NPY-fibers are present in the stria terminalis (*st*): type one are thin and run parallel to the stria terminalis, while type 2 (*orange arrowheads*) are diagonally oriented and display multiple varicosities (*white arrow* depicts an NPY neuron within the stria terminalis). **c**–**e** Dual labeling of Y2 receptors (*red*) and NPY-GFP (*green*) in the stria terminalis. Although Y2 receptors and NPY are running in parallel, they do not co-localize in individual fibers. Note the NPY-GFP labeled transverse axons with dense varicosities (**d**, *orange arrows*). *Scale bar* 200 µm

On the other hand, NPY-positive fibers were also detected within the stria terminalis, the main output tract of the amygdala (Fig. 6a, b), indicating the presence of NPY projection neurons connecting the CEm with downstream targets, such as the BNST. In the stria terminalis, NPY and Y2 receptors display a similar distribution, but the respective fibers were rather oriented in a parallel fashion than displaying real co-localization (Fig. 6c–e). An additional source of NPY was provided here by neurons within the supracapsular part of the stria terminalis (arrow in Fig. 6d). There, NPY-GFP-positive neurons were residing predominantly in the “medial and lateral pocket” extending their axons with many varicosities diagonal to the projecting fibers of the stria terminalis (arrowheads in Fig. 6d).

### Neuronal tract tracing of NPY neurons within the central extended amygdala

**Fig. 7:**
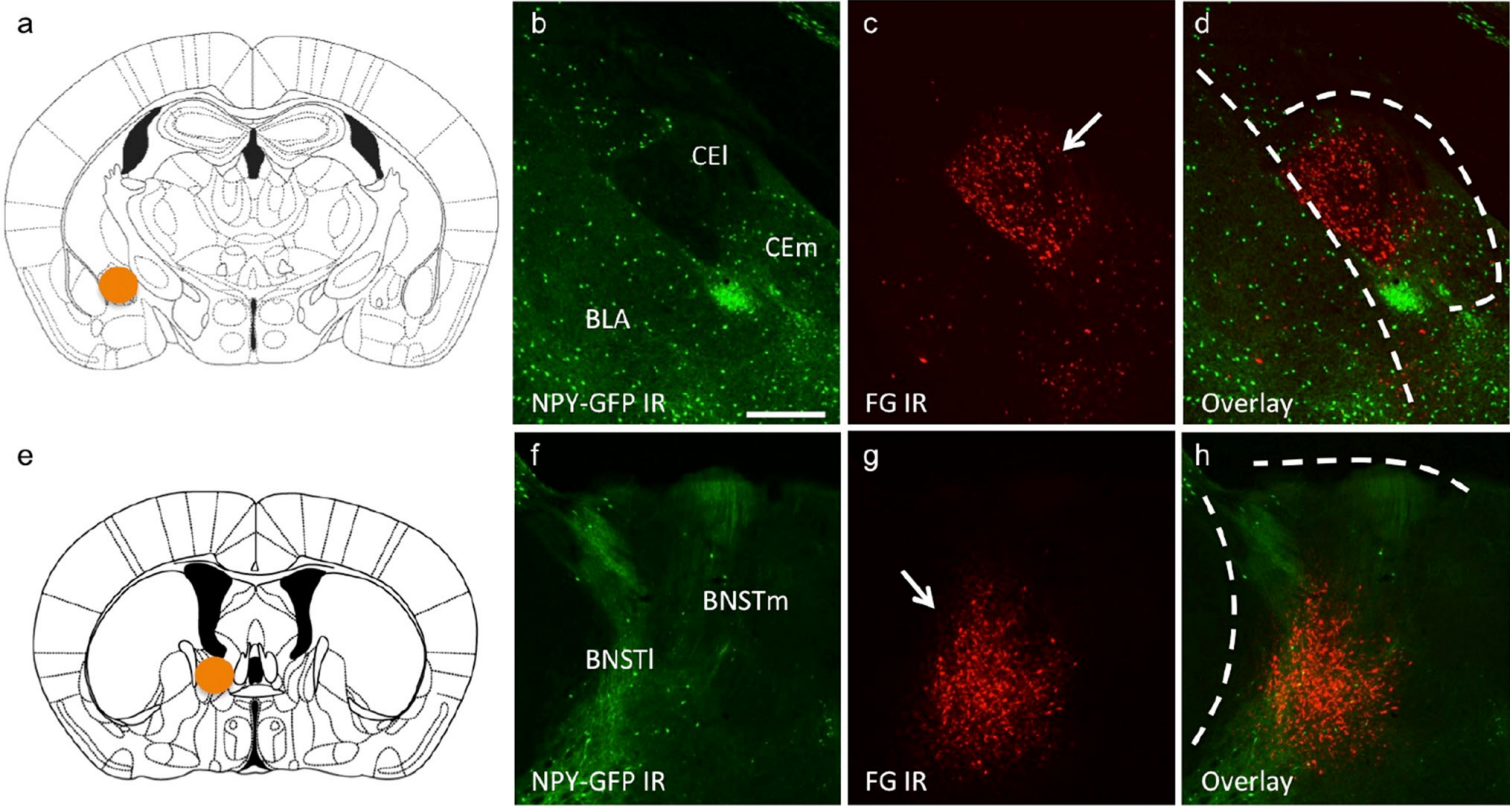
Verification of the injection site for the retrograde tracer Fluorogold in BNST and CEA injected mice. **a** Atlas image depicting the injection site for the CEA, **b** NPY-GFP fluorescence in the CEA, **c** FG fluorescence and **d** overlay showing the injection site of the retrograde neuronal tract tracer Fluorogold in a coronal section of an NPY-GFP mouse brain. **e** Atlas image depicting the injection site for FG in the BNST, **f** NPY-GFP endogenous fluorescence, **g** FG fluorescence and **h** overlay depicting the injection site in the BNST in a coronal section of an NPY-GFP mouse brain. *Scale bar* 100 µm. *Arrows* indicate injection site

**Fig. 8:**
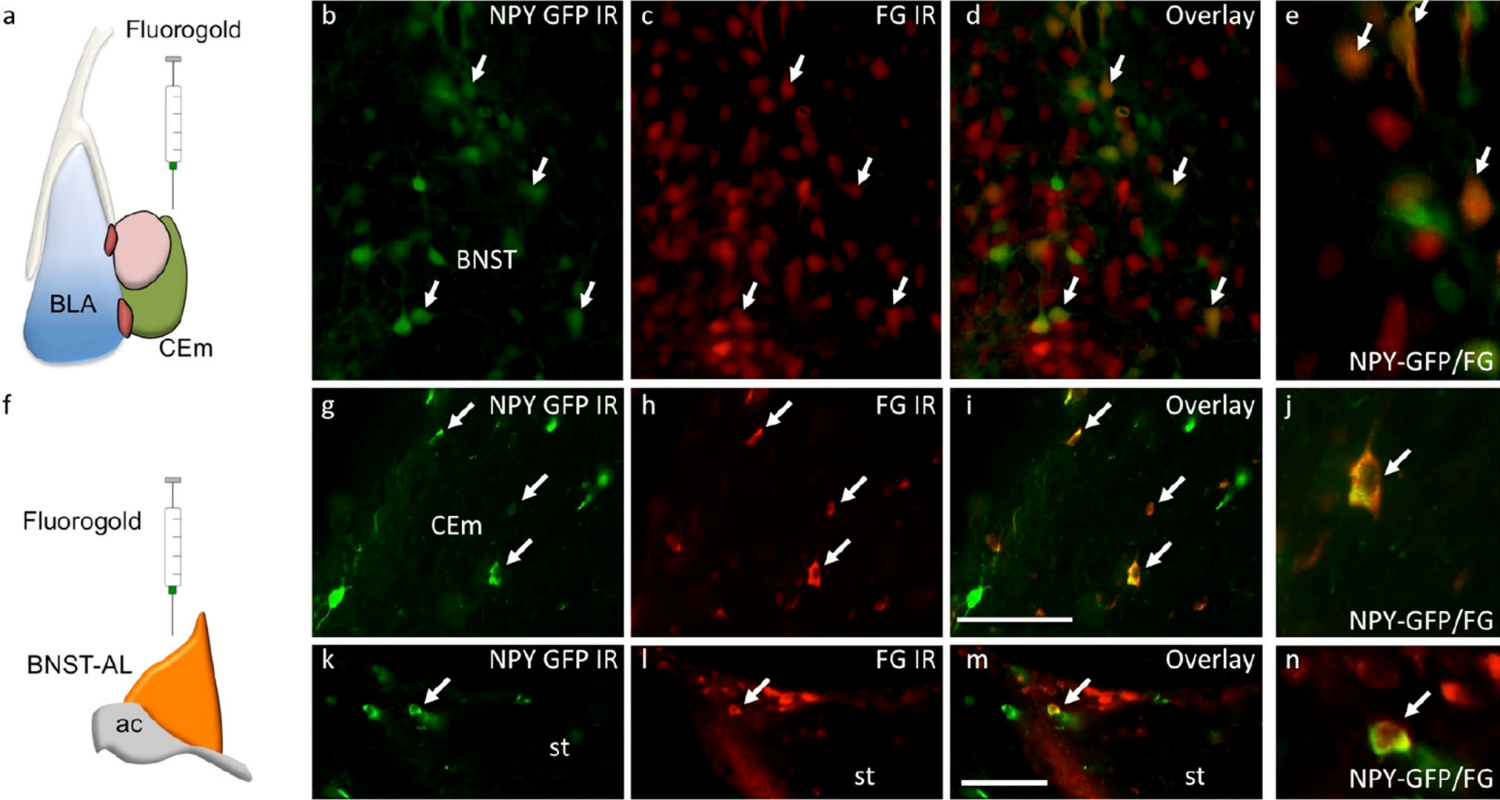
Neuronal tract tracing of NPY-expressing neurons in the central extended amygdala. **a** Injection of the retrograde tracer Fluorogold (*FG*) into the centromedial amygdala (*CEm*). **b**–**d** Dual immunofluorescence of NPY-GFP and the retrograde neuronal tracer FG in the bed nucleus of the stria terminalis (BNST) demonstrating that NPY-positive neurons are projecting from the BNST to the CEm. **e** Higher magnification image of dual-labeled cells expressing NPY-GFP and FG (*white arrows*) in the BNST. **f** Injection of the retrograde tracer FG into the BNST of NPY-GFP mice and **g**–**i** dual immunohistochemistry of NPY-GFP and FG in the CEm, demonstrating that NPY-GFP neurons project from the CEm to the BNST. **j** Higher magnification of a dual-labeled cell for NPY-GFP and FG in the CEm. **k**–**m** Dual immunofluorescence of NPY-GFP and FG in the stria terminalis (*st*), illustrating the presence of NPY neurons in the medial pocket of the st that are targeting the BNST. **n** Higher magnification image of a dual-labeled cell expressing NPY-GFP and FG in the st. *BNST-AL* anterior lateral region of the BNST. *Scale bars* **b**–**d**, **g**–**i**, **k**–**m** 100 µm and in **j** for **e**, **j**, **n** 20 µm

A subgroup of NPY neurons in the CEm co-labeled with CR (Fig. 2l–o) and both, CR and NPY were also observed in the stria terminalis (Fig. 6a–b), indicating that NPY/CR neurons of the CEA are projecting to other brain areas. To investigate in more detail the connectivity of NPY-expressing projection neurons in the central extended amygdala (CEA and BNST), we injected the retrograde neuronal tract tracer hydroxystilbamidine (Fluorogold, FG) into the CEm (Fig. 7a–d) or BNST (Fig. 7e–h) of NPY-GFP mice, followed by dual immunofluorescence for FG and NPY-GFP (Fig. 8a, f). FG was co-localized with NPY-GFP in the BNST (Fig. 8b–e) as well as in the CEm (Fig. 8g–j) after injection of FG into the CEm and BNST, respectively, suggesting that NPY-expressing neurons are projecting from the CEA to the BNST and also backwards from the BNST to the CEA (Tables 4, 5). Specifically, neuronal tract-tracing results indicated that 7.7 % (4/56) and 8.1 % (5/62) of NPY-GPF neurons in the lateral and medial BNST, respectively, project to the ipsilateral CEA. Interestingly, a minor population of NPY-GFP neurons, 1.6 % (1/64) and 4.1 % (2/49) of the lateral and medial BNST, respectively, project to the contralateral CEA (Table 4). On the other hand, 4.3 % of NPY-GFP neurons (6/139) of the CEm are projecting to the ipsilateral BNST. No projections of NPY neurons originated from the CEl and none of the NPY-GFP neurons in the CEA projected to the contralateral BNST. However, 25.8 % (8/31) of NPY-GFP neurons located within the medial and lateral pocket of the stria terminalis projected to the ipsilateral BNST (Table 5). Thus, NPY may be released within the central extended amygdala not only from local SST containing interneurons in the CEm, BNST and stria terminalis, but also from projection neurons connecting the CEm with the BNST and vice versa the BNST with the CEm.

**Table 4:** Co-localization of NPY-GFP and FG in the BNST and stria terminalis after FG injection into the CEA

Nucleus	Single-labeled NPY-GFP+ neurons	Double-labeled NPY-GFP+ neurons	Percent of NPY-GFP+ neurons double-labeled with FG
BNST lateral ipsi	52	4	7.69 % (4/56)
BNST lateral contra	62	1	1.56 % (1/64)
BNST medial ipsi	57	5	8.06 % (5/62)
BNST medial contra	47	2	4.08 % (2/49)
Stria terminalis ipsi	66	1	1.49 % (1/67)
Stria terminalis contra	69	0	0 % (0/69)

**Table 5:** Co-localization of NPY-GFP and FG in the CEA and stria terminalis after FG injection into the BNST

Nucleus	Single-labeled NPY-GFP+ neurons	Double-labeled NPY-GFP+ neurons	Percent of NPY-GFP+ neurons double-labeled with FG
Centrolateral ipsi	21	0	0 % (0/21)
Centrolateral contra	26	0	0 % (0/26)
Centromedial ipsi	133	6	4.32 % (6/139)
Centromedial contra	115	0	0 % (0/115)
Stria terminalis ipsi	23	8	25.81 % (8/31)
Stria terminalis contra	8	1	11.10 % (1/9)

### Role of Y2 receptors on inhibitory synaptic transmission in the CEm

**Fig. 9:**
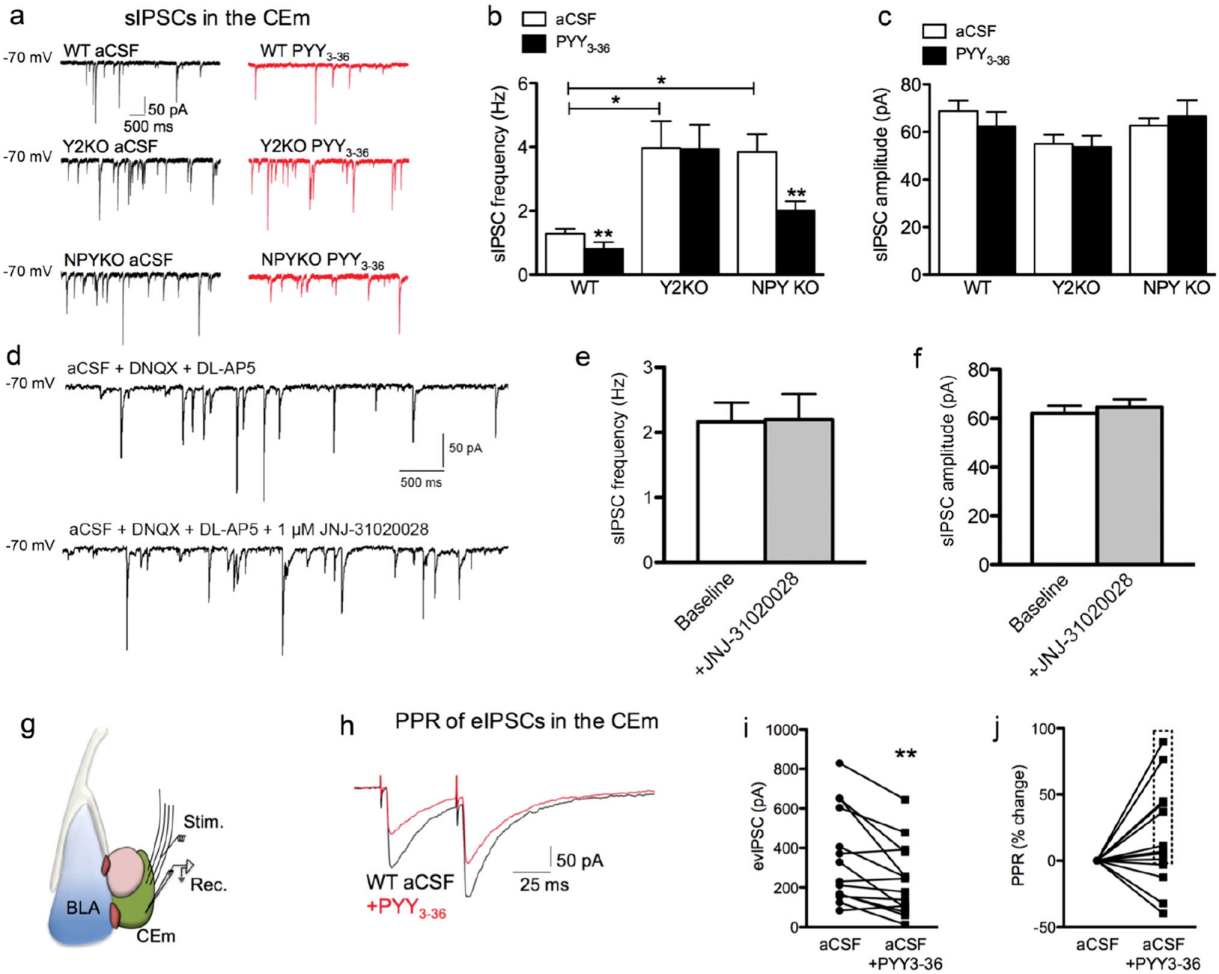
Y2 receptor activation reduces GABAergic input to CEm neurons. **a** Representative traces of sIPSCs recorded in WT, Y2KO and NPYKO mice before and after PYY_3-36_ application. **b**–**c** PYY_3-36_ reduces the frequency but not amplitude of sIPSCs (note the increased baseline frequency of sIPSCs in Y2KO and NPYKO mice compared to WT) (**paired *t* test, *p* < 0.01; *one-way ANOVA, *p* < 0.05; *n* = 5 cells from 5 slices from 3 mice WT, 6 cells from 6 slices from 3 mice Y2KO and 6 cells from 6 slices from 4 mice NPYKO). **d** Representative traces of sIPSCs recorded prior to, and after JNJ-31020028 application. **e**–**f** The Y2R antagonist, JNJ-31020028, did not alter the frequency or amplitude of sIPSCs in the CEm (paired *t* test, *p* > 0.05, *n* = 9 cells from 9 slices from 4 mice). **g** Schematic of the recording configuration. **h** Representative traces of medially evoked IPSCs in CEm neurons of WT mice before and after PYY_3-36_. **i**–**j** PYY_3-36_ reduced the amplitude of eIPSCs (paired *t* test, *p* < 0.01) but did not significantly alter the PPR, although the majority of cells revealed an increase in PPR (10 out of 14 cells, boxed area; *n* = 14 cells from 14 slices from 7 mice)

We next investigated the functional role of Y2 receptors in the CEm by recording spontaneous inhibitory postsynaptic currents (sIPSCs) before and after application of the Y2 receptor agonist PYY_3-36_ in the CEm of wildtype (WT), NPYKO and Y2KO mice. Two-way ANOVA for repeated measurements revealed a significant difference between genotypes and treatment (Fig. 9a–c, genotype: *F*_(2/14)_ = 6.69, *p* < 0.01, treatment: *F*_(1/14)_ = 23.96, *p* < 0.001 and interaction: *F*_(2/14)_ = 12.31, *p* < 0.001). In WT mice, neurons of the CEm exhibited sIPSCs with a mean frequency of 1.3 Hz that was reduced by 37 % after bath application of the Y2 receptor agonist, PYY_3-36_ (*t*_(4)_ = 4.96, *p* < 0.01; Fig. 9a, b). The amplitude of sIPSCs was unaltered (Fig. 9c). To test the specificity of PYY_3-36_ on Y2 receptors we next recorded sIPSCs in the CEm of mice lacking the Y2 receptor (Y2KO). PYY_3-36_ did not alter the frequency or amplitude of events *(p* > 0.05; Fig. 9a–c), demonstrating its specificity, but interestingly, the baseline frequency of sIPSCs was significantly higher in Y2KO mice compared to WT mice (one-way ANOVA with Bonferroni post hoc test: WT vs. Y2KO: *t*_(2)_ = 2.96, *p* < 0.05; Fig. 9a, b). To investigate this issue, we recorded sIPSCs in the CEm of NPYKO mice. Baseline sIPSC frequency was higher in NPYKO mice compared to WT, and similar to sIPSCs in Y2KO mice (one-way ANOVA with Bonferroni post hoc test: WT vs. NPYKO: *t*_(2)_ = 2.84, *p* < 0.05). Application of PYY_3-36_ significantly reduced the frequency of sIPSCs in NPYKO mice (paired *t* test: *t*_(5)_ = 6.67, *p* < 0.01; Fig. 9b) but had no effect on sIPSC amplitude (paired *t* test: *t*_(5)_ = 0.09, *p* > 0.05, Fig. 9c). We hypothesized that the increase in basal inhibition of the CEm in Y2KO and NPYKO mice may be due to either spontaneous NPY release in the slice or a result of germline deletion of NPY or the Y2 receptor. To specifically address this issue we recorded sIPSCs before and after application of the Y2 receptor antagonist, JNJ-3102002 (Fig. 9d–f). Bath application of the Y2R antagonist had no effect on the frequency (paired *t* test: *t*_(8)_ = 0.18, *p* > 0.05) or amplitude (paired *t* test: *t*_(8)_ = 0.81, *p* > 0.05) of sIPSCs.

To test the hypothesis that Y2 receptors reduce the frequency of sIPSCs in the CEm by a presynaptic mechanism, we recorded electrically evoked IPSCs (eIPSCs; Fig. 9g) in response to paired pulse stimulation of medial inputs to the CEA, thought to arise from the BNST (Delaney and Sah 2001). The amplitude of eIPSCs was significantly reduced upon application of the Y2 receptor agonist PYY_3-36_ (Fig. 9h, i), but overall we did not detect a change in the paired pulse ratio (PPR, a measure of neurotransmitter release probability). Specifically, the PPR increased in 10 out of 14 neurons, consistent with a presynaptic localization of Y2 receptors and a reduction in GABA release probability; however, four neurons exhibited a reduction in PPR (Fig. 9j) likely indicating a heterogeneous input to the CEm. Taken together, application of PYY_3-36_ in acute brain slices from WT mice demonstrates that Y2 receptor activation reduces inhibitory input to the CEm, while the increased frequency of sIPSCs observed in Y2KO and in NPYKO mice indicates that Y2 receptor stimulation during pre- or postnatal development significantly modulates inhibitory synaptic transmission.

### Role of Y2 receptors on excitatory synaptic transmission in the CEm

**Fig. 10:**
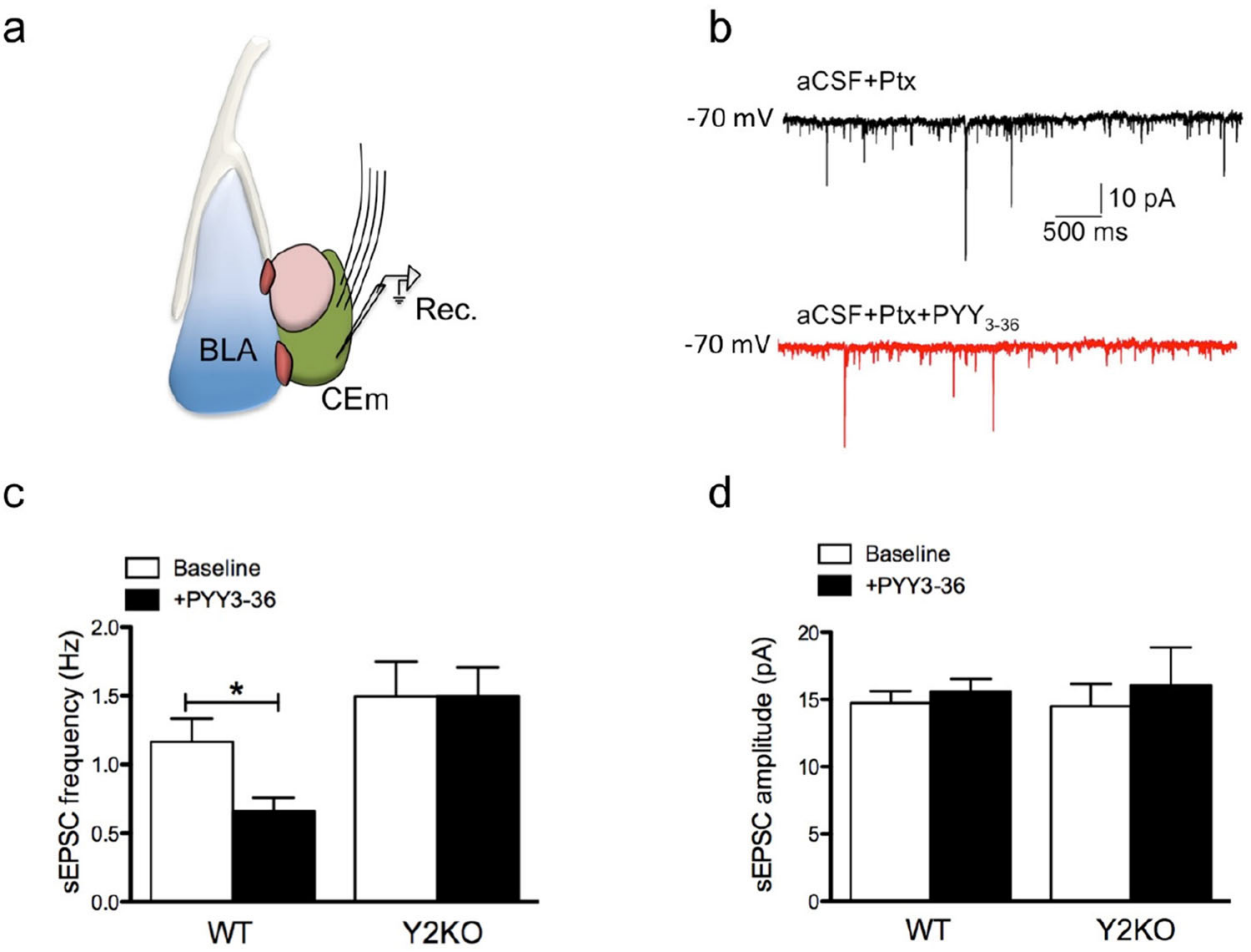
PYY_3-36_ reduces excitatory input to CEm neurons in WT mice. **a** Schematic of the recording location. **b** Representative traces of sEPSCs recorded in the presence of the GABA_A_ receptor antagonist picrotoxin (Ptx). **c**–**d** PYY_3-36_ reduced the frequency (*paired *t* test, *p* < 0.05) but not amplitude of sEPSCs in CEm neurons, and had no effect on excitatory synaptic transmission in cells recorded from Y2KO mice (*n* = 6 cells from 6 slices from 3 WT mice; 5 cells from 5 slices from 3 Y2KO mice)

Together, the absence of co-localization of NPY-GFP and Y2 receptors in the CEm (Fig. 5h–o) and the reduction of sIPSC frequency upon application of PYY_3-36_ (Fig. 9) suggest that predominantly presynaptic Y2 receptors serve as hetero-receptors that reduce inhibitory input in the CEm. However, excitatory inputs from the BLA and cortical areas also target the CEm and may therefore also be regulated by NPY acting on presynaptic Y2 receptors. To investigate the role of Y2 receptors on excitatory input to the CEm, we recorded spontaneous excitatory postsynaptic currents (sEPSCs) in CEm neurons under baseline conditions and in the presence of PYY_3-36_ (Fig. 10a)_._ As shown in Fig. 10, application of the Y2 receptor agonist PYY_3-36_ significantly reduced the frequency of sEPSCs by 29 % (paired *t* test: *t*_(5)_ = 2.94, *p* < 0.05; Fig. 10b, c) but did not alter the amplitude (Fig. 10d), consistent with the presence of presynaptic Y2 receptors on glutamatergic inputs to the CEm. No difference was detected in Y2KO mice, confirming the specificity of the compound. In contrast to our finding that sIPSC frequency was elevated in Y2KO mice, no difference in sEPSC frequency was detected in Y2KO mice under baseline conditions, suggesting that during development Y2 receptors may have an important role in fine-tuning, particularly, inhibitory synaptic transmission in the CEm.

## Discussion

Combining slice electrophysiology, neuronal tract-tracing and immunohistochemistry, we provide evidence that NPY and Y2 receptors are ideally positioned to fundamentally shape CEA output. Specifically we demonstrated that Y2 receptor activation attenuates GABA as well as glutamate release after activation by exogenously applied PYY_3-36_. Interestingly, germline deletion of Y2 receptors resulted in a baseline increase of sIPSCs but not in sEPSCs in the CEm, suggesting an important role of Y2 receptors during establishment of inhibitory synapses. Using immunohistochemistry we found that NPY is expressed by multiple populations of neurons in the CEA, BNST and Im, while the Y2 receptors are also located on afferent and/or efferent projections.

Compared to other amygdala nuclei, the highest levels of Y2 receptors are found in the CEA and BNST. These Y2 receptors may be present primarily on local circuit neurons; however, our observation of dense Y2 receptor labeling within the stria terminalis suggests that the Y2 receptor is also expressed by GABAergic, NPY-negative projection neurons. As shown previously by receptor binding, Y2 receptors are located on axon terminals of CEA neurons targeting the BNST, hypothalamus and brainstem (Tasan et al. 2010), suggesting that NPY modulates GABAergic projections originating from the CEA. In particular, the lateral part of the anterior BNST that harbors predominantly GABAergic neurons (Poulin et al. 2009) receives dense projections from the CEA (Dong et al. 2001) and is involved in the mediation of anxiolytic-like behavior (Gungor and Pare 2014; Jennings et al. 2013; Sink et al. 2011). Thus, Y2 receptors that reduce GABA release from CEA projections may modulate anxiety-like behavior by disinhibiting CEA projection targets, such as the anterior BNST. Neuropeptides are preferentially released upon strong, high-frequency stimulation. Thus, fearful situations that strongly activate NPY neurons may cause NPY release and result in a time-lagged but prolonged action of NPY limiting an otherwise excessive fear response (Heilig et al. 1994; Heilig 2004).

**Fig. 11:**
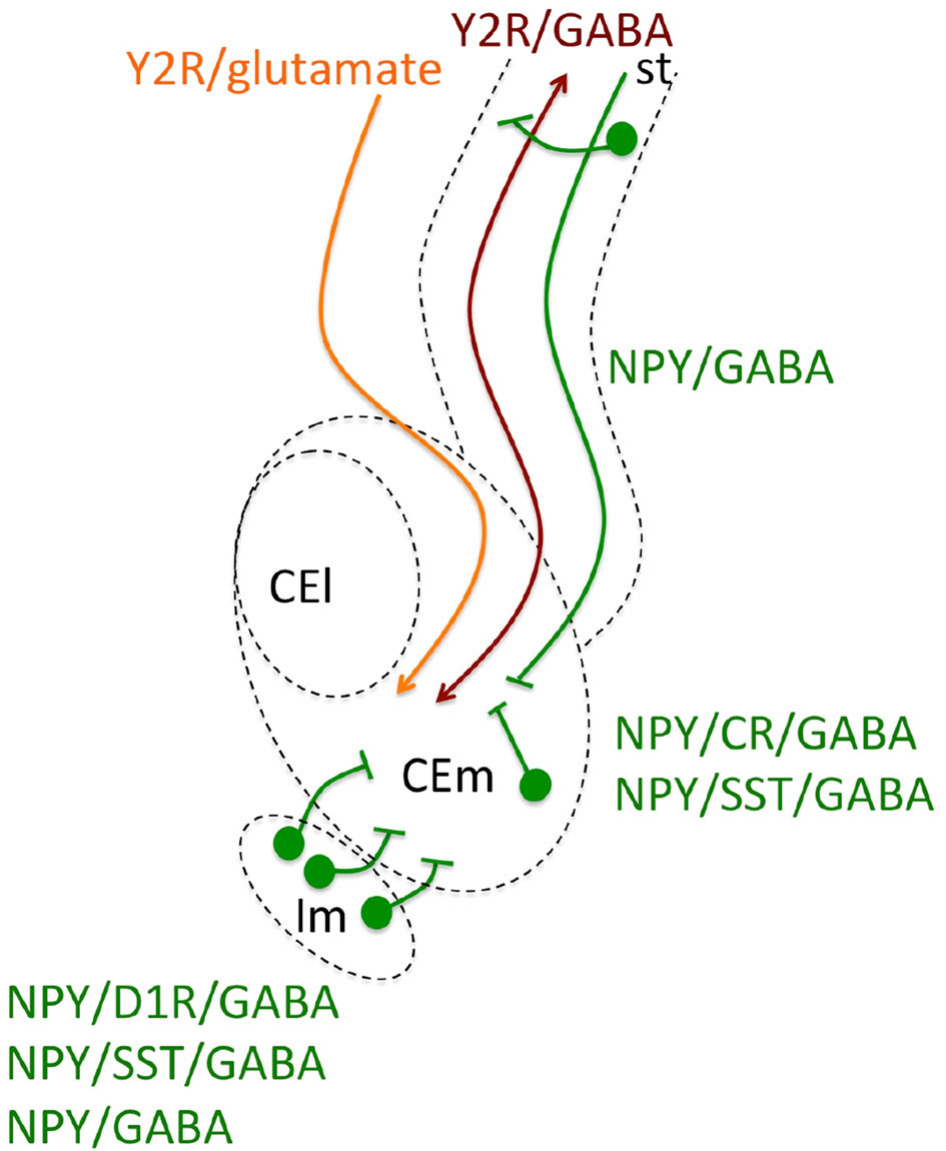
Schematic of Y2 receptor (Y2R) distribution and different sources of NPY in the central amygdala. NPY is expressed in CEm neurons (NPY/somatostatin/GABA) and in CEm afferences (NPY/GABA). GABAergic projections from NPY-expressing intercalated neurons of the Im, NPY/dopamine D1 receptor (D1R/GABA), may also be a source of NPY. The Y2 receptor is highly expressed in the CEm on both excitatory and inhibitory inputs. Thus, NPY-Y2R signaling may provide dynamic modulation of CEm activity

Several lines of evidence indicate that Y2 receptors reduce inhibition of the CEm by a presynaptic mechanism. First, application of PYY_3-36_ reduced the frequency, but not the amplitude of sIPSCs in WT and NPYKO mice. Furthermore, an increase in the PPR of eIPSCs (in 10 out of 14 cells) electrically evoked at GABAergic projections medial to the CEm, suggests that activation of presynaptic Y2 receptors reduces GABA release probability in the CEm. These CEm targeting GABAergic terminals that contain Y2 receptors and/or NPY, likely arise from different locations, such as BNST or hypothalamus. However, we did not observe immunohistochemical co-labeling of Y2 receptors and NPY, suggesting that the concept of Y2 receptors as auto-receptors (Broberger et al. 1997) regulating and limiting NPY release does not hold true for the central extended amygdala (Stanic et al. 2011). We rather propose Y2 receptors as hetero-receptors that are expressed locally and on afferent and efferent projections of the CEm, while NPY is provided by multiple sources within this system. For instance, NPY is predominantly expressed by SST-containing interneurons in the CEm and stria terminalis (Table 2). Thus, Y2 receptor containing afferent and efferent projections in the CEm are surrounded by NPY-expressing interneurons that may provide an important source of NPY for long-term suppression of synaptic activity (Fig. 11). In addition, NPY is also expressed by CEA projection neurons, BNST to CEm back-projections as well as by neurons that are located within the stria terminalis (Tables 4, 5). Some of the latter are even targeting the BNST. Both, NPY and CR were present in the stria terminalis, the main output fiber tract of the CEm, suggesting that NPY/CR neurons are projection neurons. On the other hand, SST-IR was not detected in the stria terminalis, indicating that NPY/SST neurons in the CEm are local circuit neurons. However, recent evidence suggests the existence of NPY/SST projection neurons in the basolateral amygdala of the rat (McDonald and Zaric 2015; McDonald et al. 2012). Further experiments are needed to investigate the phenotype of NPY/SST neurons also for the CEA and BNST.

Recent studies have demonstrated that Y2 receptor activation attenuates mIPSC frequency in the BNST (Kash and Winder 2006; Pleil et al. 2012). Here, we demonstrate a reduction of sIPSCs and sEPSCs in the CEm upon activation of the Y2 receptor. Interestingly, baseline sIPSCs but not sEPSCs in the CEm were equally increased in Y2KO and NPYKO mice, suggesting constitutive activation of Y2 receptors by NPY on GABAergic neurons in WT mice, a phenomenon that has been described for the mossy fiber pathway previously (Tu et al. 2005). On the other hand, bath application of the Y2 receptor antagonist JNJ-31020028 on acute brain slices of WT mice did not change sIPSCs, inconsistent with tonic release of NPY in the CEA. Rather the absence of NPY and Y2 receptors during embryonic or postnatal development may lead to a constitutive, increased activation of GABAergic, but not glutamatergic projections in the CEA. This speaks against a tonic release of NPY in the CEA, but highlights the importance of NPY and Y2 receptors during development for establishing the necessary connectivity of emotion-relevant pathways. In support of this notion, Y5KO mice are insensitive to NPY at excitatory mossy-fiber CA3 synapses (Guo et al. 2002). Further studies are warranted to elucidate this altered connectivity in NPYKO and Y2KO mice. There are certainly limitations to what extent a slice preparation can recapitulate in vivo physiology, thus further studies using in vivo electrophysiology are needed to substantiate the current data. Neuropeptides are released at relatively low concentrations and during periods of high-frequency firing, but display high receptor affinity and prolonged duration of action. NPY generally acts on different Y receptors (Y1, Y2, Y5) and may diffuse within the central extended amygdala and along the stria terminalis by volume transmission acting there on Y2 receptors potentially generating a prolonged inhibitory action by reducing the activity of long-distance fiber tracts. However, not all afferent GABAergic terminals targeting the CEm are Y2 receptor positive, as indicated by the heterogeneous response to PYY_3-36_ observed using electrical stimulation.

Interestingly, PYY_3-36_ also decreased the frequency of sEPSCs and in contrast to sIPSCs, the baseline frequency of sEPSCs was not altered in Y2KO mice. While inhibitory fibers may originate from adjacent intercalated cell masses, the BNST or from local interneurons, the origin of excitatory inputs that express presynaptic Y2 receptors is not yet clear. However, in particular, the prefrontal cortex contains significant amounts of Y2 receptor expressing neurons (Stanic et al. 2006, 2011) and targets both the CEm as well as the BNST (Bienkowski and Rinaman 2013). Future experiments are required to identify the origin of GABAergic and glutamatergic CEm afferents that are modulated by NPY and Y2 receptors.

It is important to note that PYY_3-36_ does not exclusively act on Y2 receptors, but is in fact also a potent agonist of the Y5 receptor. In this regard, Sajdyk et al. (2002) have suggested that in the basolateral amygdala of the rat Y2 receptor stimulation results in an anxiogenic phenotype, whereas activation of Y5 receptors is anxiolytic (Sajdyk et al. 2002). However, in the present study application of PYY_3-36_ on slices from Y2KO mice did not alter sIPSCs or sEPSCs, suggesting that, at least in the CEA, Y2 receptors, and not Y5 receptors lead to the observed reduction in synaptic transmission.

Previous evidence suggests that Y2 receptor stimulation promotes anxiety (Bacchi et al. 2006; Nakajima et al. 1998; Redrobe et al. 2003; Tasan et al. 2010; Tschenett et al. 2003), but see also Kask et al. (1998). However, evidence from NPY and Y receptor KO mice suggests that Y2 receptor activation may reduce expression of conditioned fear while promoting fear extinction (Verma et al. 2012). Different brain circuits are involved in fear and anxiety (Davis et al. 2010; Walker and Davis 1997; Walker et al. 2003) and Y2 receptors may play different roles in the respective circuitries. For instance, it may be possible that Y2 receptors located on glutamatergic and GABAergic terminals in the CEA differentially affect fear and anxiety-related behaviors, respectively. However, addressing this, and similar concepts will require further investigation.

In summary, we have identified multiple populations of NPY-expressing neurons within or adjacent to the central extended amygdala (Fig. 11). While NPY is predominantly expressed by SST-expressing neurons, we have identified several other important NPY-expressing neuronal subtypes. These include, CR-expressing neurons of the CEm, D1 receptor expressing neurons of the Im and SST-expressing neurons in the Im. Furthermore, we provide evidence that NPY neurons reciprocally connect the CEm with the BNST and some of these are located within the stria terminalis. Lastly, we have shown that NPY acting on Y2 receptors modulates inhibitory and excitatory signaling in the CEm.

## Electronic supplementary material

Below is the link to the electronic supplementary material.

Supplementary material 1 (DOCX 214 kb)
