# Supplementary material for: Structure and function of the amygdaloid NPY system: NPY Y2 receptors regulate excitatory and inhibitory synaptic transmission in the centromedial amygdala
Source: Brain Struct Funct. 2015 Sep 13;221(7):3373–91. doi: 10.1007/s00429-015-1107-7 (PMC4696156; doi:10.1007/s00429-015-1107-7)
Supplement: Supplementary file 1 — Supplementary material 1 (DOCX 214 kb) [file 429_2015_1107_MOESM1_ESM.docx]

**
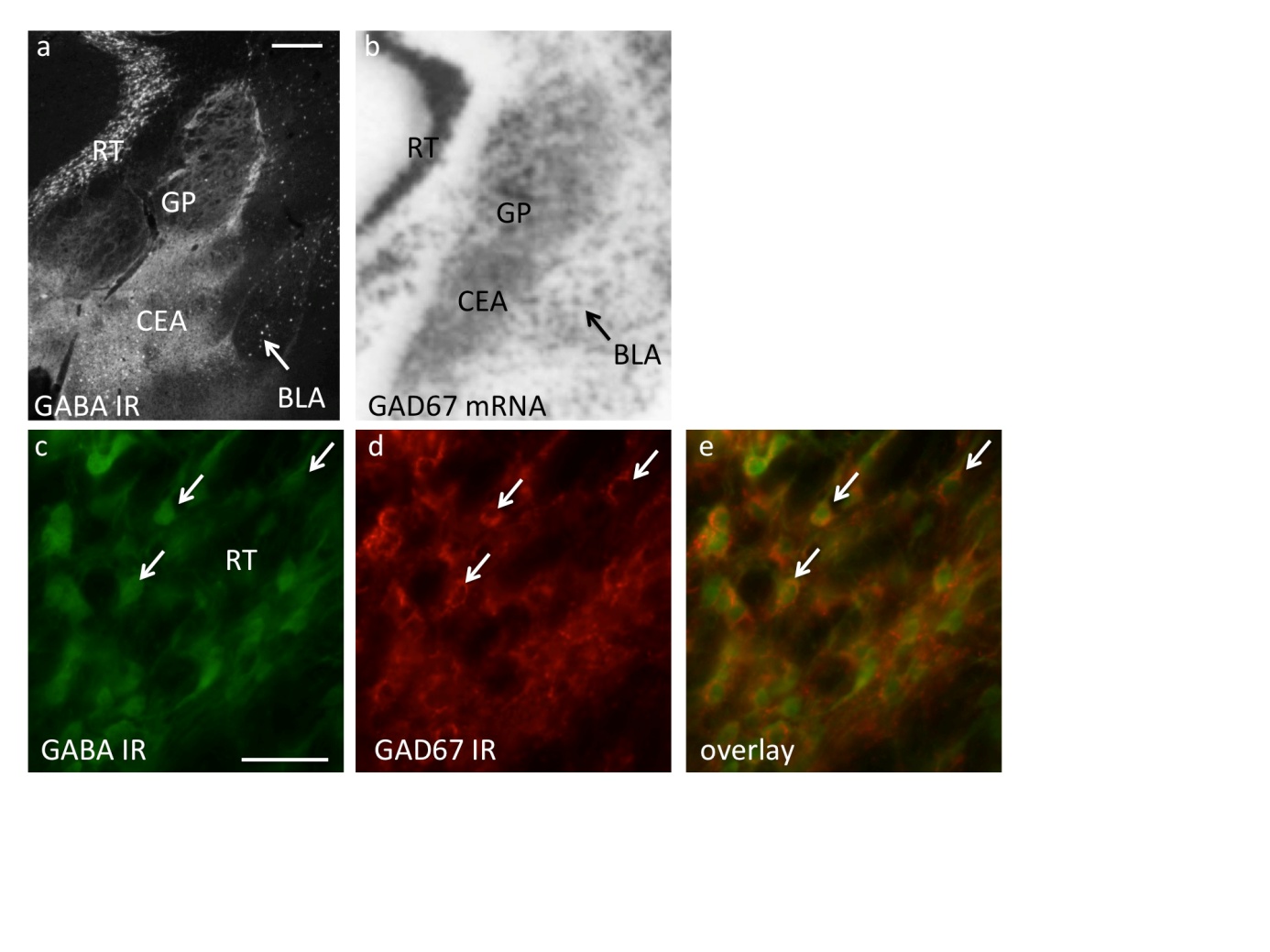
**

**Supplementary figure 1.** Validation of GABA immuno-labeling procedure. (a) Photomicrograph of a GABA immunohistochemistry on a coronal section of a mouse brain compared to (b) autoradiograph of an *in situ* hybridization for the GABA-synthesizing enzyme GAD67 displays congruent distribution. (c) Dual-immunohistochemistry for GABA and (d) GAD67 in the reticular thalamic nucleus (RT) with (e) extensive co-localization demonstrating the immuno-labeling of GABA-ergic cells (Note that the expression of GAD67 is predominantly in fibers while the GABA antibody also labels cell bodies, an important prerequisite for co-localizing immunohistochemical markers to specific cell populations). Scale bars (a-c) 500µm, (c-e) 100µm.
